# Of Mice and Women: A Comparative Tissue Biology Perspective of Breast Stem Cells and Differentiation

**DOI:** 10.1007/s10911-015-9341-4

**Published:** 2015-08-19

**Authors:** Gabriela Dontu, Tan A. Ince

**Affiliations:** Stem Cell Group, Breakthrough Breast Cancer Research Unit, Research Oncology, King’s College London School of Medicine, 3rd Floor Bermondsey Wing, Guy’s Hospital, London, SE1 9RT UK; Sylvester Comprehensive Cancer Center, Braman Family Breast Cancer Institute, Interdisciplinary Stem Cell Institute and Department of Pathology, University of Miami Miller School of Medicine, 1501 NW 10th Ave., Miami, 33136 FL USA

**Keywords:** Stem cells, Mammary gland, Comparative pathology, Hormone receptors

## Abstract

Tissue based research requires a background in human and veterinary pathology, developmental biology, anatomy, as well as molecular and cellular biology. This type of c*omparative tissue biology* (***CTB***) expertise is necessary to tackle some of the conceptual challenges in human breast stem cell research. It is our opinion that the scarcity of CTB expertise contributed to some erroneous interpretations in tissue based research, some of which are reviewed here in the context of breast stem cells. In this article we examine the dissimilarities between mouse and human mammary tissue and suggest how these may impact stem cell studies. In addition, we consider the differences between breast ducts vs. lobules and clarify how these affect the interpretation of results in stem cell research. Lastly, we introduce a new elaboration of normal epithelial cell types in human breast and discuss how this provides a clinically useful basis for breast cancer classification.

## Introduction

Integration of experimental results from multiple species and correlating these with human disease pathology is a multidisciplinary challenge [1]. In the case of normal breast stem cell research, this challenge includes correlating results obtained in mouse models with human tissues [2]. Furthermore, the insights gleaned from studying normal cellular lineages must be related to disease states [3, 4]. This integration process has not been always successful, partly due to lack of communication between different fields of research, decreasing number and lack of involvement of CTB experts [1, 5–8]. Here, we examine some of the factors that contribute to these challenges.

### Human versus Mouse Models in Mammary Stem Cell Biology

Several rigorous experiments demonstrated that a single normal mouse mammary stem cell can re-generate an entire glandular tree capable of producing milk in five serial transplantations [9]. Such an experiment fulfills the most stringent *in vivo* test for the identification of oligopotential stem cells in the mouse mammary gland.

For obvious reasons related to size, an entire human mammary gland or even an entire mammary lobe cannot be generated in the mouse mammary fat pad. In xenotransplantation experiments, human cells generate at best the equivalent of a very small mammary terminal duct unit, but no primary or secondary ducts, and they do not repopulate the entire fat-pad. So far, successful xenotransplantation cannot be achieved from single human mammary cells.

To date, the lowest number of human mammary epithelial cells implanted in the humanized clear fat pad of immunodeficient mice that generated outgrowths was ten cells, representing the mammosphere initiating cells [10]. In the same study by Pece et al., the lowest number of cells prospectively isolated from normal breast tissue which generated outgrowths when implanted *in vivo* was 500 cells. These represented the *in vivo* equivalent of mammosphere initiating cells [10].

One should note, however, that the *in vivo* outgrowths of human cells only form ducts right around the implantation site and do not form a complete ductular tree across the fat-pad like the mouse cells. What are the reasons for the failure of a single human cell to repopulate the entire mouse mammary fat-pad? One answer might be that there are no oligopotential stem cells in the adult human breast. Alternatively, it is possible that the correct oligopotential cell subpopulation has not been isolated so far. A third possibility is that cross-species differences between human and mice may not permit such an experiment to succeed. In this section we will consider the latter two possibilities with a particular attention to comparative tissue biology and hormonal states.

**Fig. 1:**
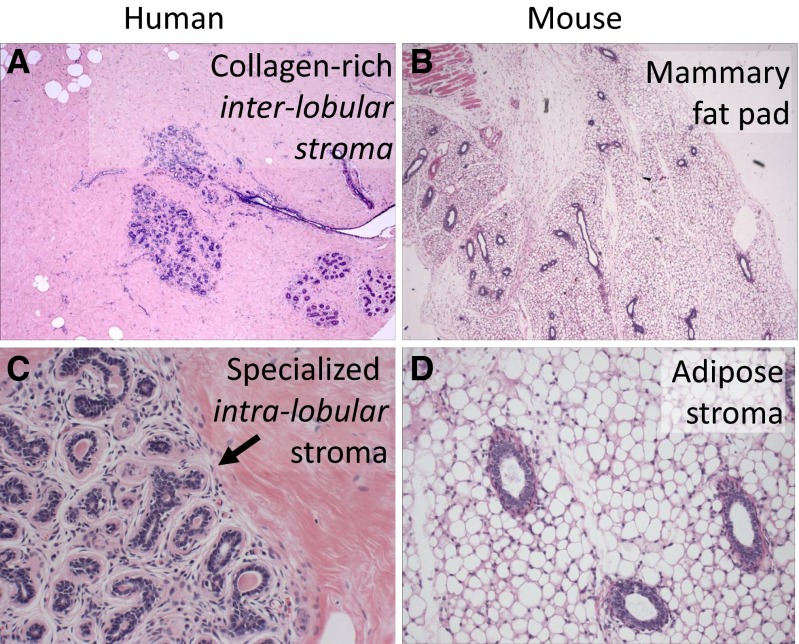
Differences in the microanatomy of human vs. mouse breast. **a** Normal human breast section. The solid pink background highlights the dense stroma with mature collagen surrounding ducts and lobules (Hematoxylin-eosin (H&E) stain of formalin-fixed paraffin-embedded (FFPE), 40×). **b** Normal mouse breast lacks the dense stromal background. Most of the tissue is composed of adipose cells (H&E stain of FFPE, 40×). **c** The arrow points to the interface between the outer dense collagenous inter-lobular stroma and the inner intra-lobular specialized stroma in human breast (H&E stain of FFPE, 400×). **d** Normal mouse breast ducts are directly surrounded by the adipose tissue, without a stromal interface. Also note that there is not a well-formed lobular structure with branching TDLU as seen in the human lobule in panel C (H&E stain of FFPE, 400×)
- There are significant differences between rodent and human hormonal milieu.
  1. The baseline systemic plasma estrogen hormone levels are up to ten-fold lower in rodent compared to primates [15].
  2. Ovulation is accompanied by a significant hormonal spike in primates that is not seen in rodents [15–17].
  3. The mouse mammary fat pad is predominantly composed of adipose cells with a scarce component of fibroblasts (Fig. 1d) [14]. In contrast, the specialized stroma of human breast immediately surrounding the TDLU contains abundant fibroblasts with distinct surface antigens, and secrete enzymes and cytokines that have a morphogenetic role in different development stages of the breast. [18, 19] The comparative representation of these specialized fibroblasts with paracrine effects in the human breast stroma is significantly higher than the mouse mammary stroma. (Fig. 1c) [14].
  4. Some of the species-specific mouse cytokines may not interact with human receptors.
  5. As described above, the human breast is very heterogeneous. In contrast, mouse mammary gland maturation is generally much more uniform and synchronized.
- There are significant differences between rodent and human reproductive cycles.
  Due to the litter size and gestational cycle differences, there is a much greater demand on the mouse mammary gland to produce milk compared to the human mammary gland. Mice have an 18–20 days gestation cycle and an average litter of 10–12 offspring. Each pup weighs 0.5–1.5 g at birth and reaches 10–12 g by the time it is weaned, around 3 weeks of age. The gestation cycle resumes 2–5 days after weaning. If we extrapolated this to human physiology, it would equal nursing ten babies that reach half the body weight of their mother in less than a month and potentially repeating this cycle every 2 months. Therefore, mouse mammary stem cells may be more robust and may have a higher regenerative capacity compared to their human counterparts.

- There are significant differences between the architecture of rodent versus human breast.
  1. The mouse mammary gland is a network of ducts ending in stem-cell enriched structures called terminal end buds (TEBs), which drive further duct elongation and branching in subsequent developmental stages.
    In contrast, the human mammary gland has a more complex structure, consisting of 17–30 individual lobes, each of them connected to the nipple. Lobules emerge through side-branching from the big ducts, to which they are connected through secondary ducts. Lobules have been classified in three types depending on maturity and branching complexity plus an additional fourth type, seen only in the lactating mammary gland, which contains alveoli filled with milk [11, 12]. The development of the human mammary gland is not synchronous. Lobules of all three types can be seen in adjacent positions in relation to the primary ducts. Entire lobes may be excluded from lactation, having only undeveloped lobules. The functional unit of the mammary gland is a collection of ductules in the composition of the lobules, the terminal ductal lobular unit (TDLU). Although it has been proposed to be the functional equivalent of the TEB in the mouse mammary gland, it has a different structure and it is not clear if it is enriched in stem or progenitor cells.
  2. The intra-lobular stroma of the human breast lobule, referred to as ‘specialized stroma’, is absent in mice. This stroma is cellular and it is typified by ‘loose’ collagen mixed with hyaluronin and other matrix proteins that envelope human TDLU. The entire TDLU structure is surrounded by dense extra-lobular stroma that is not as cellular as the intra-lobular stroma; it is predominantly composed of dense collagen that forms a thick layer between the TDLU and surrounding adipose tissue (Fig. 1a). In contrast, mouse mammary gland is mostly composed of adipose tissue that directly juxtaposes ducts without a significant matrix layer (Fig. 1b).
  3. There is extensive baseline branching in the resting TDLU of human breast (Fig. 1c) [13]. In contrast, baseline mouse mammary tree is predominantly unbranched (Fig. 1d) [14].
  4. After cessation of lactation the TEB of the mouse mammary gland reverts back to a baseline morphology with few branches. In contrast, human breast TDLU remains extensively branched after lactation. The impressive level of involution seen in mouse breast after pregnancy and lactation is not observed in human breast to the same extent [14].

The differences between rodents and human listed above suggest that expecting a single human mammary stem cell to re-populate the entire mouse mammary fat-pad may not be realistic. The failure to do so may not be evidence against presence of oligopotential human breast stem cells. However, it is also worth remembering that bona fide human stem cells - hES or iPS - can form all three germ layers in teratoma-like structures in mice [20]. Intriguingly, cells from normal human breast with hES like multipotential differentiation capacity have been isolated and these cells are capable of forming mammary outgrowths capable of lactation upon xeno-transplantation [21]. In conclusion, given all these cross-species differences we should not take absence of evidence for evidence of absence regarding the existence of oligopotential adult human breast stem cells capable of forming an entire gland.

### Heterogeneity of Normal Human Breast Samples

Another variable one should be mindful about is the source of the ‘normal’ breast tissue used for research. The vast majority of normal breast tissue for research comes from cosmetic mammary reduction surgeries. This is a self-selected patient subpopulation that is not representative of the larger population. Typically, patients of younger age are under-represented and overweight patients are over-represented and Asian patients have no representation. The second source is the ‘normal’ tissue adjacent to a tumor or from the contralateral tumor-free breast. A third source of tissue are prophylactic surgeries in BRCA mutation carrier patients or patients with DCIS. The concern for the two latter categories is that such tissue may be ‘*tumor*-*free*’ but not ‘normal’.

The profile of the normal cells in breast tissues originating from cosmetic, prophylactic and therapeutic surgeries may not be identical. The breast anatomy dictates that superficial regions close to the nipple will have larger ducts and fewer lobules. In contrast, tissue from deeper regions will have fewer ducts and more lobules. It is worth pointing out that the entire mammary gland is removed in prophylactic surgeries (BRCA), whereas reduction mammoplasties are generally subtotal. It is not difficult to envision that sampling bias such as deep vs. superficial tissue or concurrent contralateral tumor may play a significant role in the apparent discordant results between different laboratories. In addition, age and pregnancy have been shown to induce changes in the DNA-methylation of mammary epithelial cells and affect their phenotype and functionality [22]. Unfortunately, in many cases these characteristics, as well as menstrual cycle, menopausal, reproductive and previous chemo or radiation treatment history are poorly reported in normal breast stem cell studies. To address some of these problems normal human breast tissue can be obtained from Komen Tissue Bank, a collection with less population bias and uniform, standardized methods of tissue procurement and processing.

### FACS versus *In situ* Identification of Rare Cells

FACS isolation of cells using *cluster differentiation* (**CD**) markers has been the gold standard for stem cell research in the hematopoietic field. More recently, this approach has been used to isolate stem-enriched cell populations from solid tissues, sometimes without adequate caution. Since FACS requires single cell suspension as starting material, examination of solid tissues with FACS requires mechanical dissociation and then enzymatic digestion of the tissue, for as long as 8–15 h. at 37 °C. During this process, the proteolytic enzymes used to digest the tissue to single cells, as well as those that are liberated from the tissue, are liable to cleave off antigens, including the very stem cell markers used for FACS-enrichment. Therefore, this treatment can potentially create *pseudo*-*marker*-*low* subpopulations.

Once a single cell suspension is generated, the cells are incubated with primary and secondary antibodies (1–2 h. at 4 °C) and passed through the FACS instrument (1–4 h. at 20 °C). This process has the potential to cause changes in marker expression, antigenicity, posttranslational modifications and alterations in cellular and functional phenotype.

Furthermore, digestion of the tissue removes all the architectural and positional information, which is another draw-back associated with using FACS in solid tissues research. One consequence of this is the inability to know whether two different cell populations are intermixed together or segregated into different tissue zones. In particular, the destruction of the stem cell niche may impact on the ability of these cells to grow *in vivo*.

For these reasons, FACS should not be used as the standalone gold standard in solid tissue stem cell research without *in situ* corroboration, which can be done with immunostaining. However, it is important to point out that unlike FACS that produces quantitative data over a wide dynamic range, most standard immunostaining methods have a narrow linear range and are difficult to quantify. Emerging technology that allows for multiple fluorochrome microscopic analysis and computer-assisted quantitative cell analysis may address some of these concerns [23]. However, no single approach is ideal. Therefore, relying on a single technique and neglecting the necessity of corroborating the same results with multiple approaches has led to several important misconceptions in breast biology, as described below.

### Location, Location, Location

Tissue stem cells are generally located in a specific and highly restricted anatomical region. For example, the rapid-cycling Lgr5(+) progenitor cells in the intestinal tract are restricted to the crypt base [24]. In contrast, the slow-cycling label-retaining Bmi1(+) stem cells are located at the +4 crypt position [25, 26]. The expression of LGR5/Bmi1 is restricted to stem/progenitor cells and would be best described as *all-or-none* or *bimodal*, meaning that the differentiated cells are negative for both markers. Likewise, the CD34/K15(+) hair follicle stem cells reside in the bulge region [27]. The expression of CD34/K15 is also bimodal; only expressed in the bulge, but not in the isthmus or infundibular region [28]. In both tissues, this stem-restricted bimodal pattern is accompanied by a gradient or stochastic expression pattern of differentiation markers.

**Fig. 2:**
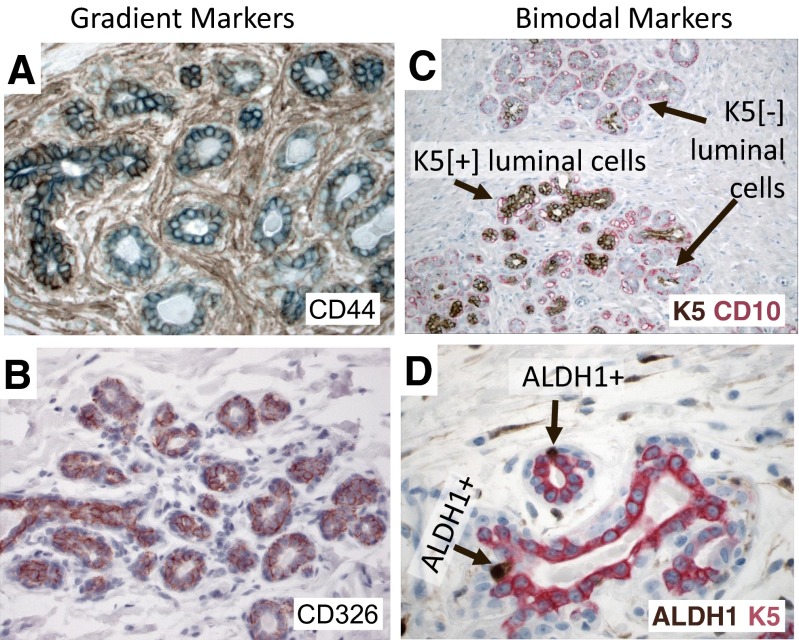
Differences in the expression pattern of various putative stem cell markers. **a** Immunostain of normal human breast section with gradient type CD44 expression (FFPE, 400×). **b** Immunostain of normal human breast section with gradient type CD326 expression (FFPE, 400×). **c** Double-immunostain of normal human breast section with bimodal keratin 5 (*brown*) and CD10 (red) expression (FFPE, 100×). **d** Double-immunostain of normal human breast section with bimodal ALDH1A1 (*brown*) and keratin 5 (*red*) expression (FFPE, 100×)

The in situ expression pattern of the putative FACS-based breast stem cell markers in normal human breast tissues does not match this well-established bimodal pattern. First, the majority of putative breast stem cell markers including CD24, CD44, CD49, CD133, CD326, EpCAM and CD10 are expressed throughout the breast, both in ducts and lobules. Second, they exhibit a gradient type expression pattern (Fig. 2a and b). Third, the cells identified by these markers alone or in complex combinations are generally not particularly rare cells. Many of the putative breast stem cell markers are expressed in most epithelial cells, albeit at different levels (Fig. 2a and b). These features are very unusual for a genuine stem cell phenotype; at least they would be unusual in any other tissue. Hence, we have to consider two possibilities: either breast epithelium is unique and disobeys the established patterns of tissue differentiation, or these CD markers are not genuine stem cell markers. Between these two possibilities the former is an exceptionalist explanation, which would require exceptional evidence that is lacking so far.

Interestingly, there are some markers that have a bimodal expression in normal breast epithelium such as K5, CD73 and ALDH1A1 (Fig. 2c). However, only ALDH1A1 and CD73 have restricted expression in rare cells [29], K5 is more broadly expressed (Fig. 2c) [29]. Intriguingly, CD73 was used to identify multi-potent stem cells with the capacity to differentiate into all three germ layers [21].

Several studies examined the potential location of the stem cells in the ductulo-lobular tree. In one study this was done by grossly dissecting ducts and lobules, and the results suggested that the stem cells are located at the junction of ducts and lobules [30]. Another study using theoretical modelling approach, concluded that the stem cells are located at end of ductules, which represent future branching points [31]. Interestingly these studies were in agreement regarding the markers that define the mammary stem cell phenotype. It was also suggested that there may be different stem cells for duct vs. lobules [30]. However, these studies have been few and far in between and more work is needed to locate the breast stem cell compartments *in situ*.

### Complete Description of Differentiated Cell Lineages: A Prerequisite to Define Stem Cells

How many subtypes of human breast cells are there? A thought experiment may help demonstrate the importance of this question for stem cell research. Let us imagine that all we knew about the cellular components of blood were presence of red and white cells. Would we be able to decipher the differentiation hierarchy of the hematopoietic cells based on this information? Thankfully, all of the differentiated cell types including B-lymphocytes, plasma cells, T-lymphocytes, neutrophils, eosinophils, basophils, mast cells, monocytes, macrophages, megakaryocytes and erythrocytes were previously described. This knowledge was essential in assessing the differentiation ability of each putative precursor cell in the hematopoietic system.

The above thought experiment should illustrate that a complete description of the differentiated cell types is a prerequisite to correctly describe differentiation lineages and putative stem cells. Yet, until recently the most rigorous functional lineage differentiation assay in breast stem cell research was the ability of a cell to give rise to luminal or myoepithelial cells, or both. This luminal vs. basal dichotomy must be replaced with a more granular description of human breast epithelial cell lineages, in order to develop a detailed differentiation hierarchy of human breast epithelium [23].

The new developments in multiplex immunostaining methods has finally allowed a more detailed description of human breast cells. In a recent study, we described eleven subtypes of normal luminal cellular states through examining fourteen markers simultaneously in nearly 15,000 normal breast cells [23, 32, 33]. In a follow-up study it was found that these cell lineages have distinct DNA methylation phenotypes, providing further evidence that they may represent different differentiation states [34]. These cell subtypes are characterized by co-expression of receptors for estrogen (**ER**), androgen (**AR**) and vitamin-D (**VDR**) and keratin 5 and grouped into four hormonal states as triple hormone receptor positive **HR3** (ER+/AR+/VDR+); double hormone receptor positive **HR2** (ER+/AR+, ER+/VDR+, or AR+/VDR+); single hormone receptor positive **HR1** (ER+, AR+ or VDR+) and triple hormone receptor negative **HR0** (ER-/AR-/VDR-) [28, 29].

**Fig. 3:**
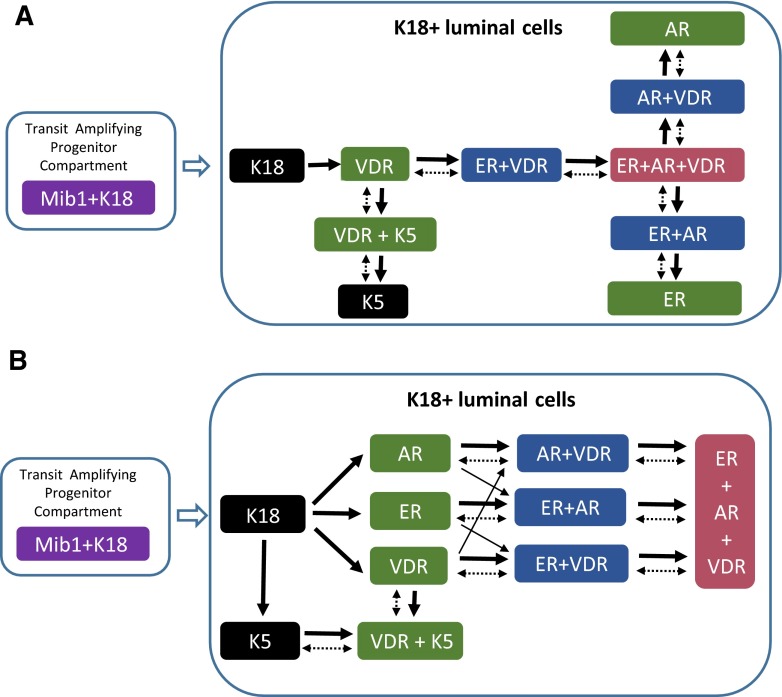
Putative differentiation steps in the luminal lineage of normal human breast epithelium. **a** The cladistic differentiation model in which only one marker can be gained or lost at each step. In addition, no more than two branches are allowed in a single step. The vast majority of the cells that are mitotically active (Mib1+/Ki67+) in normal human breast are K8/18+ cells that are negative for AR/ER/VDR/K5/K14/K17/SMA/CD10, which makes them the only candidate for transit-amplifying cells. **b** The phylogenetic differentiation model in which more than two branch points are allowed at each step. In addition, convergence into the same phenotype from multiple branches is permitted. Whether these differentiation steps are unidirectional (solid arrows) or bidirectional (dashed arrows) is not known at the moment. HR0 (black), HR1 (green), HR2 (blue), HR3 (red), transit amplifying cells (purple)

Through these recent studies it was found that only the cells that are negative for ER, AR, VDR and K5 are mitotically active, suggesting that the transit amplifying progenitors in human breast are ER (−), AR (−), VDR (−) and K5 (−) [23]. Based on this observation it was possible to imagine a putative differentiation scheme for these normal breast cell types using cladistic rules: that only one marker can be gained or lost at each differentiation step and there can be a maximum of two branches in a single step (Fig. 3a) [33]. However, alternative models of differentiation steps are possible including a phylogenetic approach permitting more than two branch points arising at each step and allowing convergence into the same phenotype from multiple branches (Fig. 3b).

**Fig. 4:**
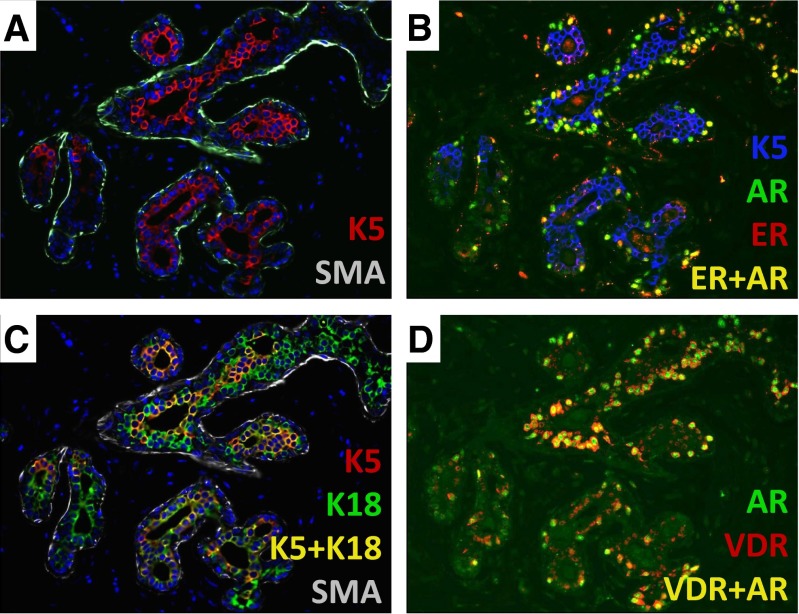
Heterogeneity of cell types in human breast. **a** Double immunostaining of normal human breast section with keratin 5 (*red*) and smooth muscle actin (SMA, *green*, FFPE, 400×). **b** Triple immunostaining of normal human breast section with keratin 5 (*blue*), estrogen receptor (*red*), and androgen receptor (*green*). The cells that co-express ER and AR are *yellow* (FFPE, 400×). **c** Triple immunostaining of normal human breast section with keratin 5 (*red*), keratin 18 (*green*) and CD10 (*white*). The cells that co-express K5 and K18 are *yellow* (FFPE, 400×). **d** Double-immunostaining of normal human breast section with AR (*green*) and vitamin-D receptor (*red*). The cells that co-express AR and VDR are *yellow* (FFPE, 400×)

An important criterion in the selection of the fourteen lineage markers was bimodal expression pattern associated with clear positive and negative cell populations *in situ* (Fig. 4) [23]. One insight from this study was the impressive heterogeneity of cellular differentiation states in the human breast (Fig. 4) [34]. It is clear that the currently available FACS-based putative stem cell markers that have a gradient type expression in situ (Fig. 2) would be difficult to use for isolating cell subtypes with a bimodal in situ distribution (Fig. 4). Thus, discovery of new stem cell markers with a bimodal in situ distribution is needed in order to correlate stem cell populations with hormonal states. Whether these thirteen cell types represent intermediate differentiation steps within a lineage or define distinct lineages remains to be seen. Other important cell types may yet have to be discovered. However, it is well known that the ligands for ER, AR and VDR are powerful regulators of differentiation and play a critical role in the development of breast tissue. Thus, as opposed to the CD marker based cellular classification, a hormone receptor based differentiation hierarchy might allow us to connect the local, systemic and environmental hormonal cues with cellular lineages and stem cell differentiation.

### What’s in a Name?

Some erroneous assumptions are difficult to correct despite repeatedly being shown to be inaccurate. One example of such a persistent misconception is the belief that breast ductal and lobular carcinomas initiate in the ducts and lobules respectively.

Cheatle et al., discussed ductal carcinomas of the breast as early as 1906 [35–37]. In an article published in 1941 Foote and Stewart defined a new entity, which they named *lobular carcinoma in situ* (LCIS) [38]. They described LCIS as a “*cancer originating in lobules*” as opposed to comedo-carcinoma, which they defined as a “*disease of the larger duct system*” [38]. Two decades later, in 1962, Johnson et al., referred to ‘noninfiltrative comedo-carcinoma’ as *ductal carcinoma in situ* (DCIS) and wrote “*it is generally conceded that carcinoma of the breast takes origin from either ducts or lobules*” [39].

Today, surgical pathologists are taught that Foote et al., were incorrect. This is based on a series of seminal articles written by *Wellings and Jensen* et al., in the early 1970s [14, 40–44]. They demonstrated, through exhaustive and comprehensive study of entire whole-mounts of breasts from nearly 200 patients, that nearly all human breast cancers initiate in the lobules, with the exception of rare papillomas [40, 42–45]. Their work showed that all of the precursor lesions such as usual hyperplasia, atypical hyperplasia, ductal and lobular carcinoma *in situ* are almost exclusively seen in the lobules first and not in ducts. This observation has been confirmed many times by other investigators [32].

Unfortunately, the important work of Wellings et al., has been largely ignored outside pathology. Hence, the assumption of ductal origin of breast cancer persists among a considerable number of basic researchers, with unintended but important consequences described below.

### Luminal vs. Basal Carcinoma

Sometimes the distinctions discussed here are dismissed as *semantic*, unjustifiably so when they result in misdirection of research efforts, including a search for the origin of breast cancer in the ducts. Furthermore, the mistaken notion of a ductal origin of human breast cancer appears to have fed into new misconceptions. In 1988 Dairkee et al., described a subgroup of triple negative breast carcinomas (**TNBC**) that express K14 [46] and had a poor prognosis [47]. They proposed that these cancers originate in “*basally located precursor cells*” and added “*it is possible*, *therefore*, *that they represent tumors of the undifferentiated basal stem cell. Interestingly*, *clinical follow*-*up of these patients suggests that these are a more aggressive group of tumors*” [46, 47]. However, others found no prognostic difference among breast cancer patients based on K5/6 and K17 expression [48].

During the early 2000s, a subset of TNBCs were found to have high levels of K5/14/17 mRNA expression in gene expression arrays. Because of the earlier work by Dairkee et al., erroneously suggesting that keratins 5, 14 and 17 are exclusively found in the normal myoepithelial/basal layer of ducts in the normal breast, these tumors were eventually referred to as basal-like carcinomas [49–52]. This appears to have led to the misconception that while ER+ and HER2+ breast cancers initiate in the luminal layer, the TNBC basal-like subtype originates in the basal layer of the breast.

**Fig. 5:**
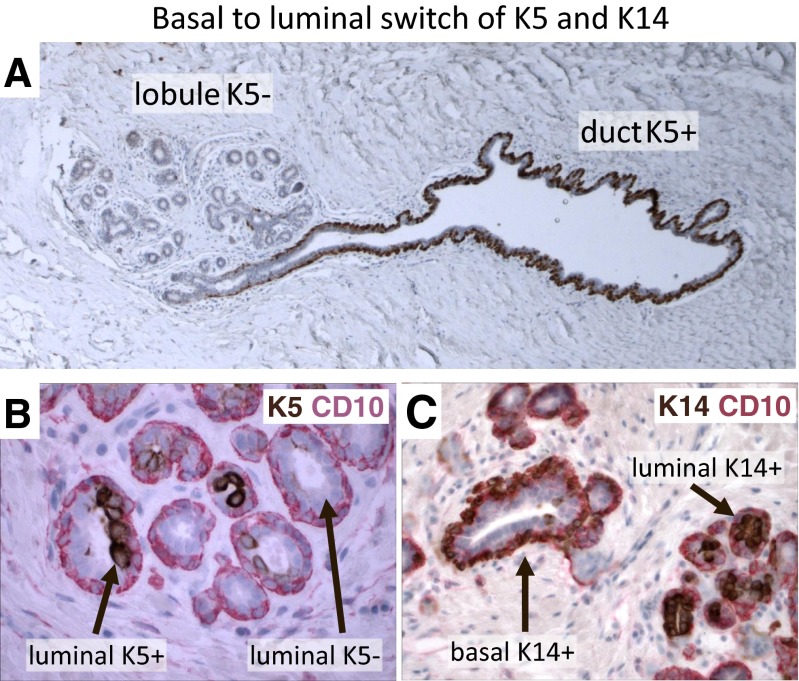
Differences in marker expression between breast ducts and lobules. **a** Immunostaining of normal human breast section with keratin 5 (*brown*). The myoepithelial cells are predominantly K5(+) in the duct. Most myoepithelial cells are K5(-) in the lobule (FFPE, 100×). **b** Double immunostaining of normal human breast section with keratin 5 (*brown*) and myoepithelial specific marker CD10 (*red*). (FFPE, 400×). **c** Double immunostaining of normal human breast section with keratin 14 (*brown*) and myoepithelial specific marker CD10 (*red*). (FFPE, 400×)

However, even before the use of basal-like carcinoma terminology became widespread [53], several investigators had shown that K5/14/17 can be expressed in the luminal layer of human breast, which was largely overlooked [54–59]. More recently, we carried out a multiplex IHC analysis of nearly 15,000 normal breast cells [23]. This study confirmed the earlier observations; it was found that K5/14/17 are predominantly expressed in the luminal layer in the lobules of normal human breast (Fig. 5) [23, 33]. Interestingly, the predominantly myoepithelial K5/14/17 expression in the large ducts gradually switches to a predominantly luminal expression in the human breast lobules (Fig. 5) [23, 33, 60]. Thus, K5/14/17 can be luminal or basal depending on the location of the cells in the human mammary ductular tree. Hence, these keratins are informative about human breast cell lineages only when the co-expression of exclusively luminal vs. basal markers are also known. In the original Dairkee et al., article the mouse monoclonal antibody 312C8-1 they used was defined as “directed towards human keratin 14” and “*reacts with basal or myoepithelial cells in the human mammary*”. Significantly, the figures that support this claim only show large ducts and not lobules, which may explain their conclusion [46].

**Fig. 6:**
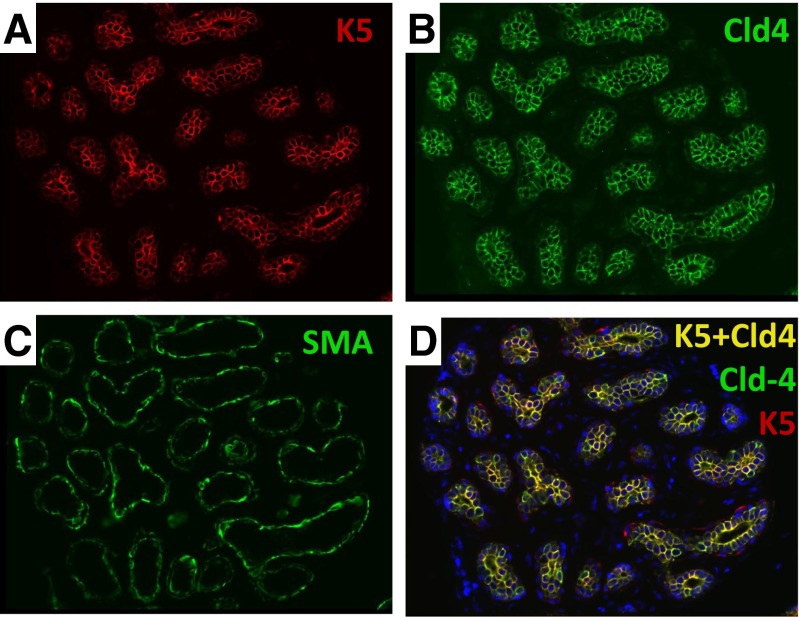
Example of a lobule entirely composed of luminal K5/Cld4 (+) cells. **a** Immunostain of normal human breast section with keratin 5 (K5, *red*, FFPE, 400×). **b** Immunostain of normal human breast section with luminal specific marker claudin4 (Cld1, *green*, FFPE, 400×). **c** Immunostain of normal human breast section with myoepithelial specific marker SMA (*green*, FFPE, 400×). **d** Double-immunostain of normal human breast section K5 (*red*) and Cld4 (*green*). The cells that co-express K5 and Cld4 are *yellow* (FFPE, 400×)

Several exclusively luminal markers have been identified in the human breast including estrogen receptor (ER), androgen receptor (AR), vitamin-D receptor (VDR), claudin-4 (Cld4) and pan-luminal keratins K7/8/18 [23, 33]. Other markers are exclusively expressed in the myoepithelial layer including smooth-muscle actin (SMA), p63 and CD10 [23, 33]. Multiplex staining studies showed that luminal K5 (+) cells co-express Cld4/K18 and they are negative for SMA/CD10 (Figs. 2, 3, 4, 5 and 6). Therefore, this expression pattern definitively confirms their luminal phenotype (Figs. 2, 3, 4, 5 and 6).

In order to determine the cell-of-origin phenotype of breast cancers, we examined the co-expression of the 14 lineage markers in nearly two thousand human breast invasive ductal carcinoma (**IDC**) samples and found that 95 % of invasive ductal carcinomas have a pure luminal phenotype including ER+, HER2+ and TNBCs [23, 33].

Based on this work, the triple negative cancers can be divided into three subgroups based on normal lineages; approximately one third of TNBCs are K7/8/18(+) and K5/14/17(−) with a straight-forward luminal phenotype [23, 33]. The remaining two thirds of TNBCs are K5 (+), which is the group that has been labeled as basal-like. However, it was found that half of these K5 (+) tumors are also VDR+/K7+/K8+/K18+ and SMA-/CD10-, as are K5 (+) normal luminal cells. Therefore the phenotype of these tumors is better described as luminal [23]. The remaining K5(+) TNBCs expresses both luminal (AR/VDR/K7/K8/K18) and myoepithelial (SMA/CD10) markers. Therefore, they are best described as ‘mixed’, luminal/myoepithelial tumors. Hence, none of the ~2,000 human breast IDCs we examined had a pure-basal like phenotype using the normal cell lineages as a benchmark [23, 33].

It is important to emphasize that we are not arguing whether basal-like carcinoma is a distinct molecular entity. There is evidence that basal-like carcinomas are molecularly different from other breast cancers [59, 61]. However; how basal-like carcinoma is defined varies greatly among different groups [49, 55, 62, 63]. Some of the confusion is caused by the well-documented discordance between mRNA vs. protein levels; it was found that approximately half of the cases that are K5/6 IHC positive are mRNA negative [64]. Thus, based on IHC they would be considered basal-like TNBC, but according to mRNA expression they would be considered luminal-like TNBC [64]. In addition, in the same study 14 % of K5/6 immunostain (IHC) negative breast cancers were found to have high K5/6 mRNA levels [64]. Thus, in more than half of the TNBCs there is discordance between what is considered basal-like depending on whether mRNA or protein based markers are used [49, 55, 59, 62, 65]. In addition, there is evidence suggesting that basal-like tumors may constitute a heterogeneous umbrella category [49, 66], harboring at least six different molecular subgroups [67–71] and five different histologic subgroups [3, 49, 55, 62, 72–75]. Nevertheless, from the perspective of cellular lineages in the normal lobule, 95 % of invasive ductal breast cancers undisputedly have a pure-luminal phenotype [23]. The remaining 5 % have a mixed luminal/basal phenotype, and none have a pure-basal phenotype. Approximately two thirds of TNBCs have a luminal phenotype and the remaining one third has a mixed phenotype [23, 33, 53].

Interestingly, the experimental mouse models as well as indirect evidence from work on human tissue also indicated that the cell-of-origin of TNBCs, basal-like carcinomas and BRCA−/− tumors are luminal cells, in agreement with the above results [76, 77]. More direct experimental evidence that the cellular origin of human breast cancer is mostly luminal comes from a study by Keller et al., in which normal mammary epithelial cells from human tissue were sorted and transformed using overexpression of oncogene combinations. Upon xenotransplantation in immunodeficient mice, transformed CD10+ myoepithelial cells generated squamous, metaplastic tumors, a subtype rarely seen in human patients, whereas transformed luminal EpCAM+ cells generated tumors with characteristics of both luminal and basal ductal carcinomas [78]. Similar conclusions were reached by Kim et al., using a different experimental approach [79].

These studies underscore that these are not semantic distinctions, because they determine which normal cells are to be studied to understand the initiation and progression of breast cancer. The name basal-like has been interpreted by some as these tumors initiating in the myoepithelial layer. As a consequence, researchers have targeted the basal cells to create TNBC models in mice and cell culture models, not so semantic considering the time and effort that has been invested in these experiments.

### Are the Cells that Co-express K5/14/17 and K7/8/18 Normal Breast Epithelial Progenitors?

The mistaken assumption that keratins K5/14/17 are never expressed in the luminal layer had consequences for normal stem cell research as well. This has led to the notion that the cells that co-express luminal keratins K7/18/19 with K5/14/17 could be breast stem cells [30, 80], because co-expression of different lineage restricted markers in the same cell has indeed been a feature of genuine stem cells in other tissues.

There is evidence suggesting that K5/K18 or K14/K18 double positive cells may be enriched for progenitors compared to other breast cell populations. However, we found that 25 % of K18(+) luminal cells are also K5 (+) (*n* = 879) and 36 % are K14 (+) (*n* = 354) on average [23, 33, 81]. In addition to these averages, some lobules are found to be entirely composed of K5/K18 double-positive luminal cells (Fig. 6) [23, 33, 58]. Such lobules have been found in all the sections that we have examined with multiplex staining. Since adult tissues cannot be composed of stem cells entirely, it is unlikely that all K5/K18(+) or K14/K18 (+) double positive cells are stem cells [82].

In contrast with these observations in the human breast, the K5 (+) or K14 (+) luminal cells are not found in the adult mouse mammary gland, which is a significant difference in the luminal cell phenotypes between these species [83]. Rare K14 (+) luminal cells are found at birth and during puberty in mice, whereas K5(+) luminal cells were not found at any developmental stage of the mouse mammary gland [83]. It appears that K5/14 are exclusively expressed in the myoepithelial layer of adult mouse breast [83]. Intriguingly, rare K6 (+) luminal mammary cells were found in adult mice [84]. Given the relative abundance of luminal K5/14 (+) cells in the adult human breast, this major difference in the spectrum of luminal cell differentiation raises further questions about extrapolation of results from mouse models to humans.

### Classification of Breast Cancers Based on Normal Lineages

**Fig. 7:**
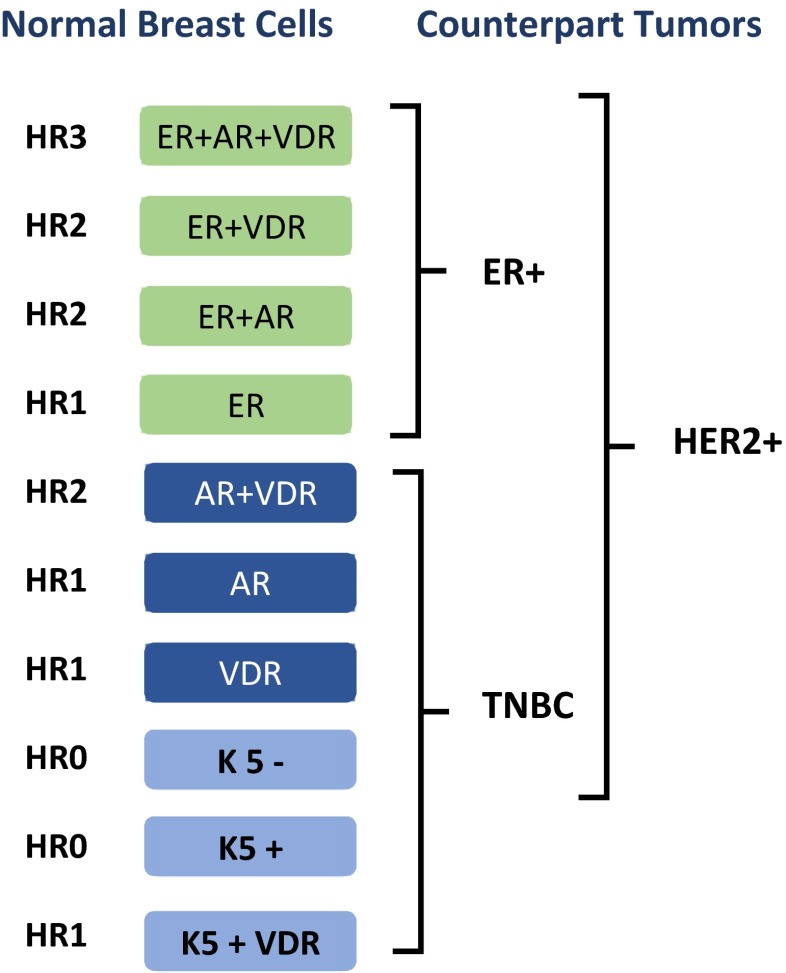
The K18 (+) luminal cell types found in normal human breast lobules and their malignant counterparts

The correct benchmark to determine cellular phenotype of human breast cancers must begin with the description of normal cell types in the lobules, where practically all human breast cancers initiate with the exception of papillomas [42, 44, 45]. As described above, the normal luminal breast cell types conform to four hormonal states based on the co-expression of ER, AR and VDR [23, 33]. The triple hormone receptor positive HR3 cells co-express ER, AR and VDR simultaneously, HR0 cells express none of the three, HR2 express ER/AR, ER/VDR or AR/VDR and the HR1 cells express a single receptor (Fig. 7) [23, 33].

When human tumors were examined for these lineages, we found that nearly all of the human tumors are similar to one of the normal cell types [23, 33]. In fact, each patient tumor was similar to one of the 10 normal cell types. This result is reminiscent of lymphomas and leukemias that resemble distinct steps in the differentiation hierarchy of normal hematopoiesis, where there is a malignant counterpart for each stage of differentiation [85–87].

Importantly, in multivariate analysis we found that that HR3 tumors have the best survival, HR1/0 tumors have the worst survival, and HR2 tumors have intermediate survival, with a relative hazard ratio of 6.9 fold between HR3 vs. HR0 tumors (*p* <0.0001) [23, 33], which is much higher than many of the traditional and molecular prognostic signatures. Hence, the normal cell type based classification revealed groups of breast cancer that have very significant clinical outcome differences.

The examination of the three standard breast cancer subtypes from the perspective of normal HR lineages also revealed some interesting insights. It was found that ~75 % of ER+ tumors have a triple positive HR3 phenotype. The remainder of ER+ tumors resemble normal HR2 ER/AR+, ER/VDR+, or HR1 ER+ cells. The TNBC tumors resemble six normal lineages including HR2 AR/VDR(+), HR1 AR(+), HR1 VDR(+), HR1 K5/VDR(+), HR0 K5(+) and HR0 K5(−) (Fig. 7) [23, 33].

Interestingly, HER2+ tumors seem to arise from all luminal lineages except those that express K5 (Fig. 7). It is intriguing to speculate whether amplification of HER2 is somehow not permissible in K5(+) luminal cells. A similar observation was made for the retinoblastoma (Rb) gene; it was found that deletion of Rb affected four of the seven cell types in the retina, but not amacrine, horizontal or glial cells [88–90]. It was later shown that Rb depletion results in formation of retinoblastomas only in cone cells but not in other retinal cells [91].

### Everything Should be as Simple as Possible, but no More

In popular culture, physicists are sometimes portrayed as fond of simpler equations that explain a phenomenon, simplicity of an answer often being thought to signify a deeper understanding. Nevertheless, as the father of one of the simplest and most powerful equations in science, Einstein said; ‘*everything should be as simple as possible*, *but not more simple*’, warning us against over-simplification.

Dichotomies such as ‘ductal vs. luminal’ or ‘basal vs. luminal’ are very seductive in their simplicity. And they have served a useful purpose to advance research in this field. However, it is increasingly becoming clear that individual tumors can have both ductal and luminal components, co-existing simultaneously [92–94]. Furthermore, the evidence reviewed here indicates that human breast has tremendous heterogeneity with many more cell types than just basal and luminal cells. Therefore, these simplistic dichotomies may have exhausted their useful life and it may be time to move beyond them.

### Comparative Tissue Biology of Breast Stem Cells

Lastly, the observations above highlight the need for involvement of comparative tissue biologists as referees in stem cell research. Over the past century several distinct disciplines of biology have emerged, such as molecular biology, cell biology, evolutionary biology, to address distinct questions utilizing specific expertise and methodology. Eventually these fields were organized into distinct academic departments or graduate programs.

It is not an overstatement to suggest that examining molecules and cells in the context of a tissue is as challenging as any other field of biology. Yet, there are no graduate programs or departments of “*tissue biology*”.

A group of research pathologists have recently pointed out the need for more formal comparative tissue biology training, in a letter entitled Do-it-yourself (DIY) pathology in Nature Biotechnology [1]:

Those of us with comparative pathology expertise have collectively noted that numerous tissue-based research studies have been published over the past decade without a pathologist among the authors, collaborators or consultants. Furthermore, based on the frequently inaccurate use of pathology terms and misinterpretation of data in many of these studies, it appears that not only the authors but also the reviewers and editors often have neglected to consult a comparative pathologist during the evaluation of such manuscripts [1].

As we have reviewed here, it is difficult to ignore DIY pathology as one of the sources of ongoing controversies in breast stem cell research. Let us not forget that it was pathologists who first put forward the concepts that constitute the current cancer stem cell model, including the hypothesis that tumorigenesis is aberrant organogenesis [95].

## Abbreviations

CTB: Comparative tissue biology
TDLU: Terminal ductal lobular unit
TEB: Terminal end bud
CD: Cluster differentiation
FACS: Fluorescent activated cell sorting
IHC: Immunohistochemistry
TNBC: Triple negative breast carcinoma
IDC: Invasive ductal carcinoma
ER: Estrogen receptor
AR: Androgen receptor
HR: Hormone receptor
VDR: Vitamin D receptor
K5: Keratin 5
LCIS: Lobular carcinoma in situ
DCIS: Ductal carcinoma in situ

## References

[1] Ince TA (2008). Do-it-yourself (DIY) pathology. Nat Biotechnol.

[2] Hollern DP, Andrechek ER (2014). A genomic analysis of mouse models of breast cancer reveals molecular features of mouse models and relationships to human breast cancer. Breast Cancer Res.

[3] Weigelt B (2012). Challenges translating breast cancer gene signatures into the clinic. Nat Rev Clin Oncol.

[4] Ince TA (2007). Transformation of different human breast epithelial cell types leads to distinct tumor phenotypes. Cancer Cell.

[5] Rodriguez-Canales J (2011). Why is it crucial to reintegrate pathology into cancer research?. Bioessays.

[6] Barthold SW (2007). From whence will they come? - A perspective on the acute shortage of pathologists in biomedical research. J Vet Diagn Investig.

[7] Bolon B (2011). Advancing translational research. Science.

[8] Valli T (2007). Over 60 % of NIH extramural funding involves animal-related research. Vet Pathol.

[9] Shackleton M (2006). Generation of a functional mammary gland from a single stem cell. Nature.

[10] Pece S (2010). Biological and molecular heterogeneity of breast cancers correlates with their cancer stem cell content. Cell.

[11] Russo J, Russo IH (2004). Development of the human breast. Maturitas.

[12] Russo L (2015). Towards understanding the molecular recognition process in prokaryotic zinc-finger domain. Eur J Med Chem.

[13] Gusterson BA, Stein T (2012). Human breast development. Semin Cell Dev Biol.

[14] Cardiff RD, Wellings SR (1999). The comparative pathology of human and mouse mammary glands. J Mammary Gland Biol Neoplasia.

[15] Chaffin CL, Vandevoort CA (2013). Follicle growth, ovulation, and luteal formation in primates and rodents: a comparative perspective. Exp Biol Med.

[16] Wu JM (2005). Ovarian aging and menopause: current theories, hypotheses, and research models. Exp Biol Med.

[17] Adashi EY (1994). Endocrinology of the ovary. Hum Reprod.

[18] Atherton AJ (1998). Differential expression of type XIV collagen/undulin by human mammary gland intralobular and interlobular fibroblasts. Cell Tissue Res.

[19] Atherton AJ (1994). Ectoenzyme regulation by phenotypically distinct fibroblast sub-populations isolated from the human mammary gland. J Cell Sci.

[20] Lensch MW, Ince TA (2007). The terminology of teratocarcinomas and teratomas. Nat Biotechnol.

[21] Roy S (2013). Rare somatic cells from human breast tissue exhibit extensive lineage plasticity. Proc Natl Acad Sci U S A.

[22] Huh SJ (2015). Age- and pregnancy-associated DNA methylation changes in mammary epithelial cells. Stem Cell Reports.

[23] Santagata S (2014). Taxonomy of breast cancer based on normal cell phenotype predicts outcome. J Clin Invest.

[24] Tian H (2011). A reserve stem cell population in small intestine renders Lgr5-positive cells dispensable. Nature.

[25] Carlone DL, Breault DT (2011). Slowly cycling versus rapidly cycling intestinal stem cells: distinct roles or redundancy. Cell Cycle.

[26] Carlone DL, Breault DT (2012). Tales from the crypt: the expanding role of slow cycling intestinal stem cells. Cell Stem Cell.

[27] Rompolas P, Greco V (2014). Stem cell dynamics in the hair follicle niche. Semin Cell Dev Biol.

[28] Rompolas P, Mesa KR, Greco V (2013). Spatial organization within a niche as a determinant of stem-cell fate. Nature.

[29] Honeth G (2014). Aldehyde dehydrogenase and estrogen receptor define a hierarchy of cellular differentiation in the normal human mammary epithelium. Breast Cancer Res.

[30] Villadsen R (2007). Evidence for a stem cell hierarchy in the adult human breast. J Cell Biol.

[31] Honeth G (2015). Models of breast morphogenesis based on localization of stem cells in the developing mammary lobule. Stem Cell Reports.

[32] Cardiff RD, Borowsky AD (2014). At last: classification of human mammary cells elucidates breast cancer origins. J Clin Invest.

[33] Santagata S, Ince TA (2014). Normal cell phenotypes of breast epithelial cells provide the foundation of a breast cancer taxonomy. Expert Rev Anticancer Ther.

[34] Houseman EA, Ince TA (2014). Normal cell-type epigenetics and breast cancer classification: a case study of cell mixture-adjusted analysis of DNA methylation data from tumors. Cancer Informat.

[35] Cheatle GL (1906). Clinical Remarks on the early recognition of cancer of the breast. Br Med J.

[36] Cheatle GL (1914). The relation between ducts and acini to cysts and cancer of the breast. Proc R Soc Med.

[37] Cheatle GL (1926). Discussion on “Pre-cancerous states.”: the pre-carcinomatous state in the breast. Proc R Soc Med.

[38] Foote FW, Stewart FW (1941). Lobular carcinoma *in situ*: a rare form of mammary cancer. Am J Pathol.

[39] Johnson J, Jackson TL, Miller W (1962). *In situ* ductal carcinoma of the breast. JAMA.

[40] Jensen HM, Rice JR, Wellings SR (1976). Preneoplastic lesions in the human breast. Science.

[41] Jensen HM, Wellings SR (1976). Preneoplastic lesions of the human mammary gland transplanted into the nude athymic mouse. Cancer Res.

[42] Wellings SR, Jensen HM (1973). On the origin and progression of ductal carcinoma in the human breast. J Natl Cancer Inst.

[43] Wellings SR, Jensen HM, DeVault MR (1976). Persistent and atypical lobules in the human breast may be precancerous. Experientia.

[44] Wellings SR, Jensen HM, Marcum RG (1975). An atlas of subgross pathology of the human breast with special reference to possible precancerous lesions. J Natl Cancer Inst.

[45] Cardiff RD, Wellings SR, Faulkin LJ (1977). Biology of breast preneoplasia. Cancer.

[46] Dairkee SH, Puett L, Hackett AJ (1988). Expression of basal and luminal epithelium-specific keratins in normal, benign, and malignant breast tissue. J Natl Cancer Inst.

[47] Dairkee SH (1987). Monoclonal marker that predicts early recurrence of breast cancer. Lancet.

[48] Potemski P (2005). Prognostic relevance of basal cytokeratin expression in operable breast cancer. Oncology.

[49] Badve S (2011). Basal-like and triple-negative breast cancers: a critical review with an emphasis on the implications for pathologists and oncologists. Mod Pathol.

[50] Sorlie T (2003). Repeated observation of breast tumor subtypes in independent gene expression data sets. Proc Natl Acad Sci U S A.

[51] Sorlie T (2001). Gene expression patterns of breast carcinomas distinguish tumor subclasses with clinical implications. Proc Natl Acad Sci U S A.

[52] Perou CM (2000). Molecular portraits of human breast tumours. Nature.

[53] Gusterson B (2009). Do ‘basal-like’ breast cancers really exist?. Nat Rev Cancer.

[54] Malzahn K (1998). Biological and prognostic significance of stratified epithelial cytokeratins in infiltrating ductal breast carcinomas. Virchows Arch.

[55] Korsching E (2008). Basal carcinoma of the breast revisited: an old entity with new interpretations. J Clin Pathol.

[56] Santini D (1996). Differentiation pathways in primary invasive breast carcinoma as suggested by intermediate filament and biopathological marker expression. J Pathol.

[57] Dairkee SH (1987). Immunolocalization of a human basal epithelium specific keratin in benign and malignant breast disease. Breast Cancer Res Treat.

[58] Boecker W, Buerger H (2003). Evidence of progenitor cells of glandular and myoepithelial cell lineages in the human adult female breast epithelium: a new progenitor (adult stem) cell concept. Cell Prolif.

[59] Lavasani MA, Moinfar F (2012). Molecular classification of breast carcinomas with particular emphasis on “basal-like” carcinoma: a critical review. J Biophotonics.

[60] Gusterson BA (2005). Basal cytokeratins and their relationship to the cellular origin and functional classification of breast cancer. Breast Cancer Res.

[61] Prat A, Perou CM (2011). Deconstructing the molecular portraits of breast cancer. Mol Oncol.

[62] Foulkes WD, Smith IE, Reis-Filho JS (2010). Triple-negative breast cancer. N Engl J Med.

[63] Carey L (2010). Triple-negative breast cancer: disease entity or title of convenience?. Nat Rev Clin Oncol.

[64] Kordek R (2010). Basal keratin expression in breast cancer by quantification of mRNA and by immunohistochemistry. J Exp Clin Cancer Res.

[65] Lusa L (2007). Challenges in projecting clustering results across gene expression-profiling datasets. J Natl Cancer Inst.

[66] Rakha EA, Reis-Filho JS, Ellis IO (2008). Basal-like breast cancer: a critical review. J Clin Oncol.

[67] Abramson VG (2015). Subtyping of triple-negative breast cancer: implications for therapy. Cancer.

[68] Chen X (2012). TNBCtype: A Subtyping Tool for Triple-Negative Breast Cancer. Cancer Informat.

[69] Lehmann BD (2011). Identification of human triple-negative breast cancer subtypes and preclinical models for selection of targeted therapies. J Clin Invest.

[70] Lehmann BD, Pietenpol JA (2014). Identification and use of biomarkers in treatment strategies for triple-negative breast cancer subtypes. J Pathol.

[71] Masuda H (2013). Differential response to neoadjuvant chemotherapy among 7 triple-negative breast cancer molecular subtypes. Clin Cancer Res.

[72] Weigelt B, Geyer FC, Reis-Filho JS (2010). Histological types of breast cancer: how special are they?. Mol Oncol.

[73] Weigelt B, Kreike B, Reis-Filho JS (2009). Metaplastic breast carcinomas are basal-like breast cancers: a genomic profiling analysis. Breast Cancer Res Treat.

[74] Weigelt B, Reis-Filho JS (2009). Histological and molecular types of breast cancer: is there a unifying taxonomy?. Nat Rev Clin Oncol.

[75] Guiu S (2012). Molecular subclasses of breast cancer: how do we define them? The IMPAKT 2012 working group statement. Ann Oncol.

[76] Molyneux G (2010). BRCA1 basal-like breast cancers originate from luminal epithelial progenitors and not from basal stem cells. Cell Stem Cell.

[77] Lim E (2009). Aberrant luminal progenitors as the candidate target population for basal tumor development in BRCA1 mutation carriers. Nat Med.

[78] Keller PJ (2012). Defining the cellular precursors to human breast cancer. Proc Natl Acad Sci U S A.

[79] Kim J (2012). Tumor initiating but differentiated luminal-like breast cancer cells are highly invasive in the absence of basal-like activity. Proc Natl Acad Sci U S A.

[80] Petersen OW (2003). Epithelial progenitor cell lines as models of normal breast morphogenesis and neoplasia. Cell Prolif.

[81] Otterbach F (2000). Cytokeratin 5/6 immunohistochemistry assists the differential diagnosis of atypical proliferations of the breast. Histopathology.

[82] Clarke CL (2004). Cytokeratin 5/6 in normal human breast: lack of evidence for a stem cell phenotype. J Pathol.

[83] Mikaelian I (2006). Expression of terminal differentiation proteins defines stages of mouse mammary gland development. Vet Pathol.

[84] Bu W (2011). Keratin 6a marks mammary bipotential progenitor cells that can give rise to a unique tumor model resembling human normal-like breast cancer. Oncogene.

[85] Shaffer AL, Rosenwald A, Staudt LM (2002). Lymphoid malignancies: the dark side of B-cell differentiation. Nat Rev Immunol.

[86] Passegue E (2003). Normal and leukemic hematopoiesis: are leukemias a stem cell disorder or a reacquisition of stem cell characteristics?. Proc Natl Acad Sci U S A.

[87] Taylor KH (2006). Promoter DNA methylation of CD10 in lymphoid malignancies. Leukemia.

[88] Bremner R (2009). Retinoblastoma, an inside job. Cell.

[89] Bremner R, Sage J (2014). Cancer: the origin of human retinoblastoma. Nature.

[90] Chen D (2004). Cell-specific effects of RB or RB/p107 loss on retinal development implicate an intrinsically death-resistant cell-of-origin in retinoblastoma. Cancer Cell.

[91] Xu XL (2014). Rb suppresses human cone-precursor-derived retinoblastoma tumours. Nature.

[92] Abdel-Fatah TM (2007). High frequency of coexistence of columnar cell lesions, lobular neoplasia, and low grade ductal carcinoma in situ with invasive tubular carcinoma and invasive lobular carcinoma. Am J Surg Pathol.

[93] Abdel-Fatah TM (2008). Morphologic and molecular evolutionary pathways of low nuclear grade invasive breast cancers and their putative precursor lesions: further evidence to support the concept of low nuclear grade breast neoplasia family. Am J Surg Pathol.

[94] Tazaki E (2013). Histopathologcial and clonal study of combined lobular and ductal carcinoma of the breast. Pathol Int.

[95] Sell S (2004). Stem cell origin of cancer and differentiation therapy. Crit Rev Oncol Hematol.

